# CDK5 Regulatory Subunit-Associated Protein 1-Like 1 Gene Polymorphisms and Gestational Diabetes Mellitus Risk: A Trial Sequential Meta-Analysis of 13,306 Subjects

**DOI:** 10.3389/fendo.2021.722674

**Published:** 2021-10-14

**Authors:** Xiang-yuan Yu, Li-ping Song, Shu-dan Wei, Xiao-lan Wen, Da-bin Liu

**Affiliations:** ^1^ Department of Epidemiology and Health Statistics, Guilin Medical University, Guilin, China; ^2^ Fujian Key Laboratory of Women and Children’s Critical Diseases Research, Fujian Maternity and Child Health Hospital, Fuzhou, China

**Keywords:** gestational diabetes mellitus (GDM), polymorphism, meta-analysis, trial sequential analysis (TSA), false-positive report probability

## Abstract

**Objectives:**

The CDK5 regulatory subunit-associated protein 1-like 1 (*CDKAL1*) contributes to islet β-cell function and insulin secretion by inhibiting the activation of CDK5. The current studies on the relationship between *CDKAL1* polymorphisms rs7756992 A>G and rs7754840 C>G and the risk of gestational diabetes mellitus (GDM) have drawn contradictory conclusions.

**Materials and Methods:**

A meta-analysis with a fixed- or random-effects model was conducted to estimate the correlation between studied *CDKAL1* polymorphisms and GDM risk with the summary odds ratio (OR) and 95% confidence interval (CI). In addition, trial sequential analysis (TSA) and false-positive report probability (FPRP) analysis were performed to confirm the study findings.

**Results:**

A total of 13,306 subjects were included in the present study. Meta-analysis results showed that the variant heterozygous and homozygous genotypes of the two polymorphisms were associated with increased GDM risk in comparison with the wild-type AA genotype (AG *vs.* AA: OR = 1.23, 95% CI = 1.08, 1.41, *p* = 0.002; GG *vs.* AA: OR = 1.47, 95% CI = 1.05, 2.05, *p* = 0.024 for rs7756992; and CG *vs.* GG: OR = 1.36, 95% CI = 1.13, 1.65, *p* = 0.002; CC *vs.* GG: OR = 1.76, 95% CI = 1.37, 2.26, *p* < 0.001 for rs7754840). The TSA confirmed a significant association between rs7754840 and the susceptibility to GDM because the cumulative Z-curve crossed both the conventional cutoff value and the TSA boundaries under the heterozygote and homozygote models.

**Conclusions:**

This study supported the finding that rs7756992 and rs7754840 are associated with susceptibility to GDM. However, further functional studies are warranted to clarify the mechanism.

## Introduction

Gestational diabetes mellitus (GDM), defined as an abnormal glucose tolerance onset or first recognition in pregnancy (1), is approximately 1% to 22% of all pregnancies (2, 3). Clinical and epidemiological studies show that GDM can lead to short- and long-term adverse consequences for both mothers and their offspring, including increases in the incidence of gestational hypertension, spontaneous abortion, respiratory distress syndrome of newborns, or the development of complex diseases such as type 2 diabetes mellitus (T2DM) and cardiovascular disease (4–7). GDM has become a major challenge in the field of public health.

It is known that pregnancy at an older age, obesity, immune status, individual nutrition, behavior, etc., are factors associated with GDM (8–11). However, these factors do not completely explain the pathogenesis of GDM. Now, there is new evidence that the risk of GDM in individuals with family history of T2DM is significantly higher and that the risk of T2DM will increase in GDM patients in the future (10, 11). It suggests that inherited genetic factors also contribute to the genesis of GDM. The single-nucleotide polymorphism (SNP) is one of the main forms of heritable variation in the human genome DNA sequence, which determines the basis of genetic susceptibility to human disease. At present, SNPs have become the best genetic marker to interpret genetic susceptibility and predict disease risk. Replication of a recent genome-wide association study (GWAS) confirmed that T2DM-associated genetic variants (*TCF7L2* rs7903146, *CDKAL1* rs7756992 and rs7754840, *MTNR1B* rs10830962, and *FTO* rs8050136) are associated with the risk of GDM (12). Functional studies show that these diabetogenic genes and polymorphisms might participate in abnormal glucose metabolism, insulin resistance, and impaired β-cell function, etc., which could lead to the occurrence and development of GDM.

The CDK5 regulatory subunit-associated protein 1-like 1 (CDKAL1) is located at the human chromosome 6p22.3, encoding a 65-Ku CDKAL1 protein. CDKAL1 is mainly expressed in human pancreatic islet cells and shows considerable homology with CDK5RAP1, a well-known inhibitor of CDK5 activation. CDK5 has been suggested to downregulate insulin secretion through the formation of p35/CDK5 complexes (13, 14). In addition, CDK5 transduces glucose toxicity signals in pancreatic β-cells (13). Given that GDM has some common risk factors and genetic susceptibilities with T2DM (15, 16), a series of studies have investigated the relationship between *CDKAL1* polymorphisms rs7756992 and rs7754840 and the risk of GDM. However, because of the limitations of the sample size and statistical efficiency of a single study, the conclusions are still inconsistent. In addition, to our knowledge, if the number of participants is less than required, under a realistic intervention effect, the constant application of a 5% statistical significance threshold would lead to too many false-positive and/or false-negative conclusions. Thus, a single meta-analysis usually does not reflect whether it has enough power to detect or refute the effect of an intervention. To get a clearer picture of the relationship between polymorphisms rs7756992 and rs7754840 of *CDKAL1* and GDM risk, we therefore performed a trial sequential analysis (TSA) and false-positive report probability (FPRP) analysis in this meta-analysis.

## Materials and Methods

### Literature Search Strategy

Published studies of *CDKAL1* rs7756992 and/or rs7754840 and the risk of GDM were searched in PubMed, Web of Science, the China National Knowledge Infrastructure (CNKI), and Wanfang databases, with languages limited to English and Chinese. The latest search was performed on March 31, 2021. The search terms “CDK5 regulatory subunit associated protein 1-like 1,” “*CDKAL1*,” “GDM,” “gestational diabetes mellitus,” “variation,” “polymorphism,” and “SNP” were used as retrieval words. Studies were also identified by a manual search of the references cited in the retrieved studies.

### Inclusion and Exclusion Criteria

Inclusion criteria were as follows: 1) original study on the association between rs7756992 and/or rs7754840 polymorphisms and the risk of GDM and 2) case–control study or GWAS with independent data, 3) with genotype and/or allele distribution information, and 4) with sufficient data for calculating an OR with its corresponding 95% CI.

Exclusion criteria were as follows: 1) duplicate data, 2) no specific genotype or allele data, and 3) family-based study, review, systematic review or meta-analysis, case report, comment, and editorial.

### Data Extraction and Literature Evaluation

Two professional investigators (X-YY and L-PS) independently searched articles and extracted target data from the included studies. If there were any dispute, it was settled by X-YY and L-PS. Data on the first author, year of publication, country, diagnostic criteria, source of controls, number of cases and controls, mean age, mean body mass index (BMI), genotype, and/or allele distribution were extracted.

In the present study, the Newcastle-Ottawa Scale (NOS) was used to assess the quality of target studies (17). The score range of the scale was from 0 (the lowest) to 9 (the highest), studies with a score <5 were considered to be of low quality, and those ≥5 to be of high quality. If a study is considered to be of low quality, it will not be included in the subsequent meta-analysis.

### Statistical Analysis

The ORs with their corresponding 95% CIs were used to assess the strength of association between the polymorphism and GDM risk. The *I* ^2^ index was calculated to quantify the degree of heterogeneity across studies, and a corresponding *p* less than 0.1 was considered significant (18, 19). The potential source of heterogeneity across studies was explored by stratification and meta-regression analysis. Begg’s tests (20) were used for testing the publication bias, and *p* < 0.05 was considered statistically significant. Sensitivity analyses were also done to assess a single study on the pooled OR. All analyses were performed by using Stata software, version 12.0 (Stata Corp LP, College Station, TX, USA).

In addition, FPRP was estimated to assess the robustness of findings of statistically significant associations. The FPRP threshold and the prior probability were set to 0.2 and 0.1, respectively, to detect the noteworthiness for OR of 1.5 (0.67 for an OR less than 1.0), with an alpha level equal to the observed *p*-value. An FPRP less than 0.2 was considered as a noteworthy association (21, 22).

### Trial Sequential Analysis

Meta-analysis might be affected by the increased risk of random errors and repeated significance testing (23). TSA can increase the robustness of the conclusions by estimating the amount of the required information size (RIS) and the threshold for statistical significance (24). During the analysis, the significance levels for type I and type II errors were set to 5% and 20%, respectively, and relative risk reduction (RRR) was set at 20%. When the cumulative Z-curve crosses the TSA boundary or enters the insignificance area, it demonstrates a sufficient level of evidence, and no further study is necessary (25). The TSA software (version 0.9.5.10 beta) was used for data processing.

## Results

### Characteristics of the Included Studies

**Figure 1:**
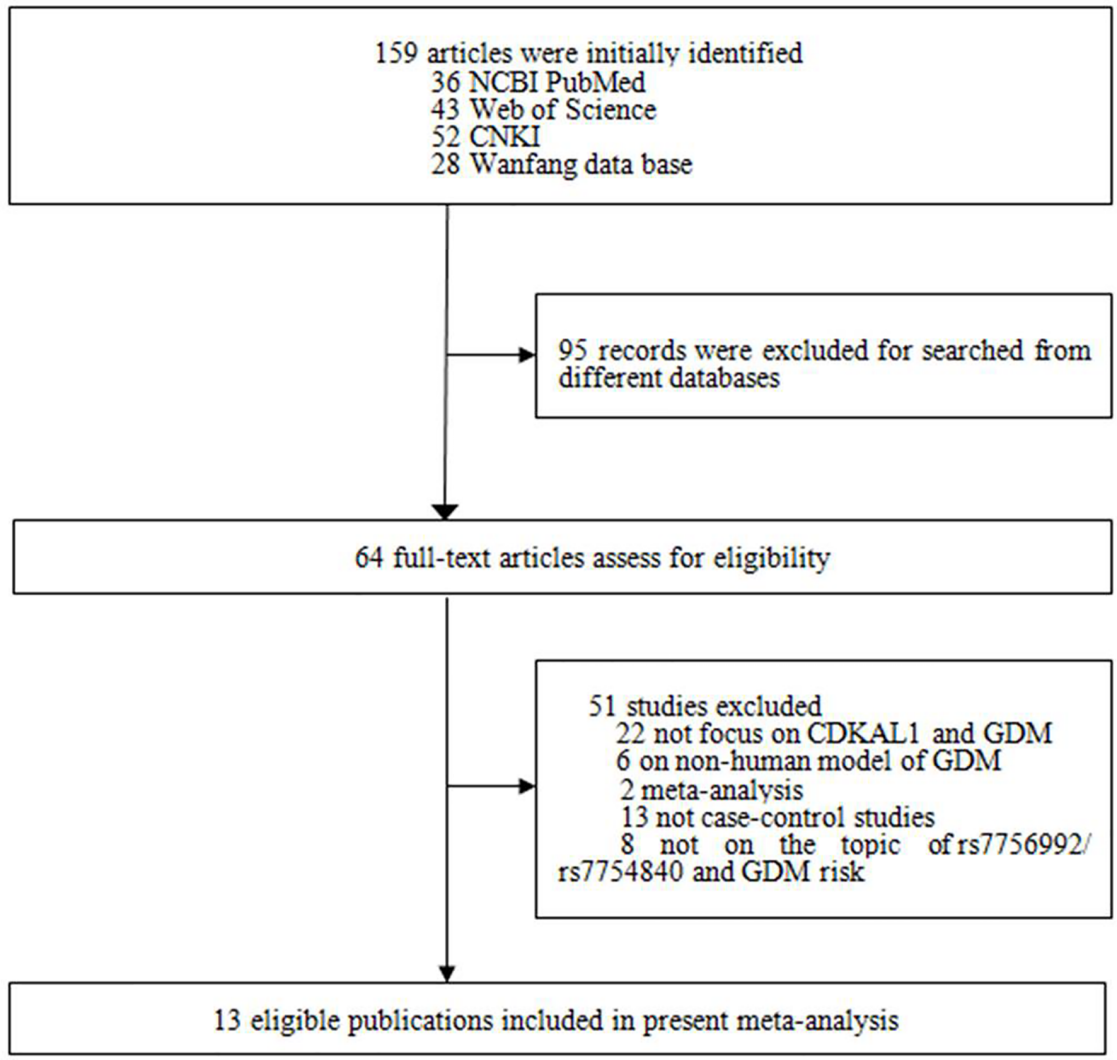
Flow chart of the process of identification of eligible studies.

The literature search and selection are shown in **Figure 1**. A total of 159 studies were initially identified, and 95 articles retrieved from different databases were excluded. Subsequently, an additional 51 studies were excluded for the following reasons: 1) 22 studies were not about *CDKAL1* gene and GDM; 2) six articles concerned a non-human model of GDM; 3) 15 were not case–control or GWASs; and 4) eight studies did not focus on the topic of *CDKAL1* rs7756992/rs7754840 and the risk of GDM. According to the study selection strategy, 13 studies (nine in English and four in Chinese) met the research theme, of which 10 were considered to be of high quality (score ≥5) and the other three [Hu et al. (26), Wu et al. (27) and Noury et al. (28)] were of low quality (score <5), as shown in **Figure 1** and **Table 1**. Finally, 10 studies of high quality with 5,094 GDM patients and 8,212 controls were ultimately recruited.

**Table 1:** Characteristics of included studies.

Author	Year	Country	Control	No. of case/control	RAF of case/control		Mean age of case/control	Mean BMI of case/control	NOS score
					rs7756992	rs7754840			
Kwak SH-a (12)	2013	Korea	Non-diabetic	468/1,242	–	0.576/0.445	31.5 ± 4.0/59.1 ± 5.6	23.3 ± 3.2/24.6 ± 3.2	8
Kwak SH-b (12)	2013	Korea	Non-diabetic	931/783	–	0.556/0.467	32.5 ± 4.0/66.1 ± 7.5	25.0 ± 4.7/23.9 ± 3.2	8
Cho YM (29)	2008	Korea	Non-diabetic	863/345	0.610/0.536	0.576/0.475	32.0 ± 3.9/64.4 ± 3.3	23.1 ± 3.6/23.9 ± 3.3	9
Lauenborg J (30)	2009	Denmark	Healthy	275/2,339	0.535/0.543	–	43.1/45.2	28.9/25.0	8
Zhang LH (31)	2009	China	NGT	471/589	0.535/0.543	0.454/0.453	32.3 ± 3.8/31.0 ± 3.8	22.4 ± 3.4/21.1 ± 2.7	8
Aris NKM (32)	2010	Malay	Healthy	174/114	0.440/0.333	0.382/0.270	29.7 ± 4.7/28.5 ± 3.6	–	5
Wang Y (33)	2011	China	NGT	697/1,020	–	0.471/0.444	32.0/30.0	21.72/21.48	8
Deng ZF (34)	2013	China	Healthy	160/160	0.572/0.582	0.159/0.041	32.1 ± 3.4/31.9 ± 2.8	25.9 ± 3.4/25.4 ± 3.2	7
Hu YH (26)	2014	China	Healthy	176/185	–	0.469/0.341	29.5 ± 4.2/28.3 ± 3.9	–	4
Kanthimathi S (35)	2015	India	NGT	495/910	0.291/0.221	0.273/0.219	28.1 ± 5.0/26.3 ± 5.5	27.5 ± 2.4/23.3 ± 4.7	6
Wu YL (27)	2015	China	Healthy	153/180	–	0.448/0.447	28.9 ± 3.6/28.1 ± 4.1	23.3 ± 2.1/22.5 ± 1.9	4
Popova PV (36)	2017	Russia	Healthy	278/179	–	0.353/0.310	31.8 ± 4.8/29.4 ± 4.8	25.7 ± 5.9/22.9 ± 4.5	5
Rosta K (37)	2017	Hungary/Austria	NGT	287/533	–	0.329/0.300	–	–	5
Noury AE (28)	2018	Egypt	NGT	47/51	–	0.660/0.618	–	–	4

NGT, normal glucose tolerance; RAF, risk allele frequency; BMI, body mass index; NOS, Newcastle-Ottawa Scale.

### Association Between rs7756992 and Gestational Diabetes Mellitus Risk

In total, six high-quality studies containing 2,376 GDM cases and 4,458 controls explored the relationship between rs7756992 and the risk of GDM (29–34). The results of meta-analysis showed that compared with the wild-type homozygous AA genotype, the variant AG heterozygous and GG homozygous genotypes were significantly associated with increased risks of GDM (AG *vs.* AA: OR = 1.23, 95% CI = 1.08, 1.41, *p* = 0.002; GG *vs.* AA: OR = 1.47, 95% CI = 1.05, 2.05, *p* = 0.024).

**Figure 2:**
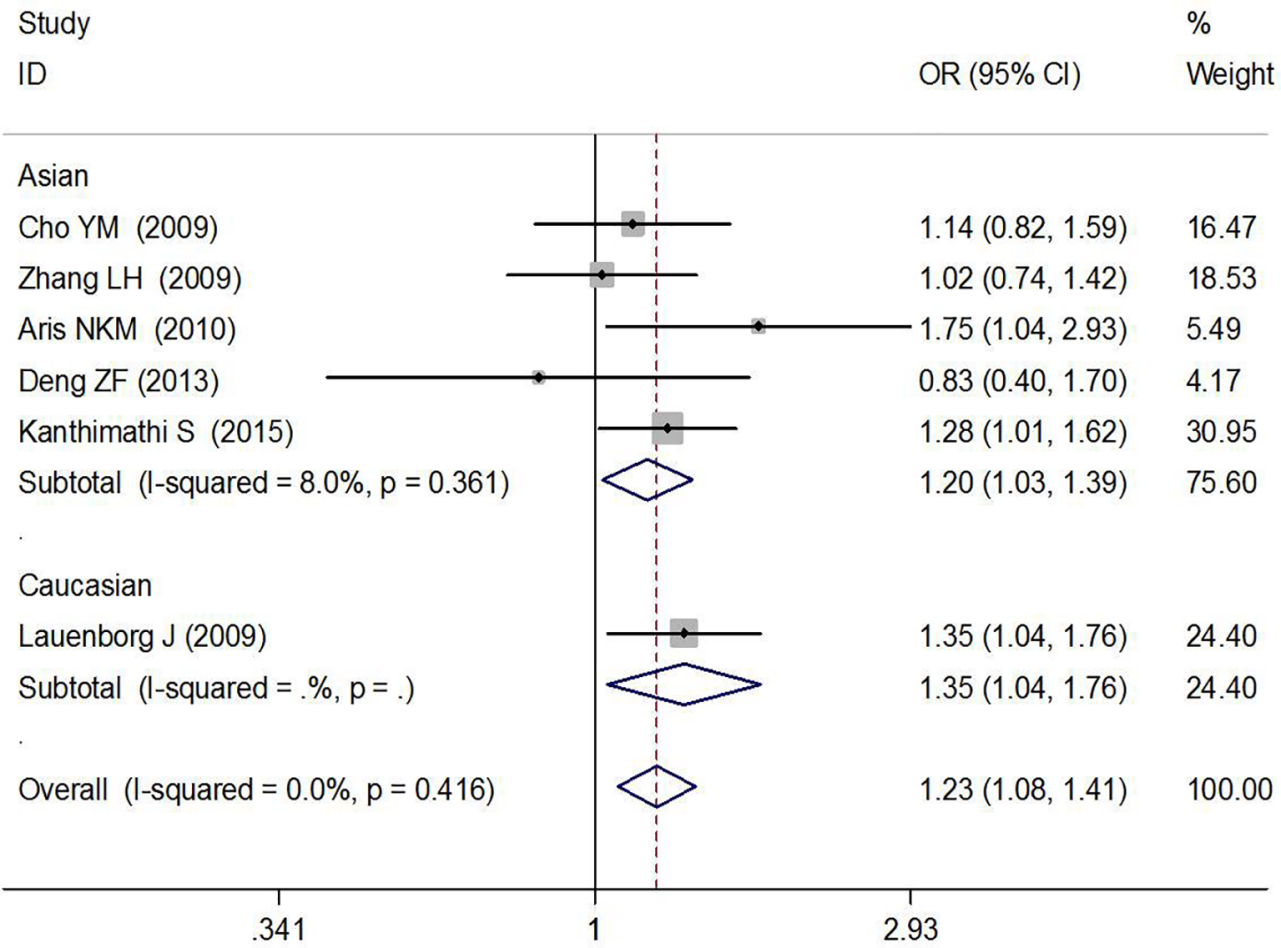
Forest plot risk of GDM associated with the *CDKAL1* rs7756992 (AG *vs.* AA).

**Figure 3:**
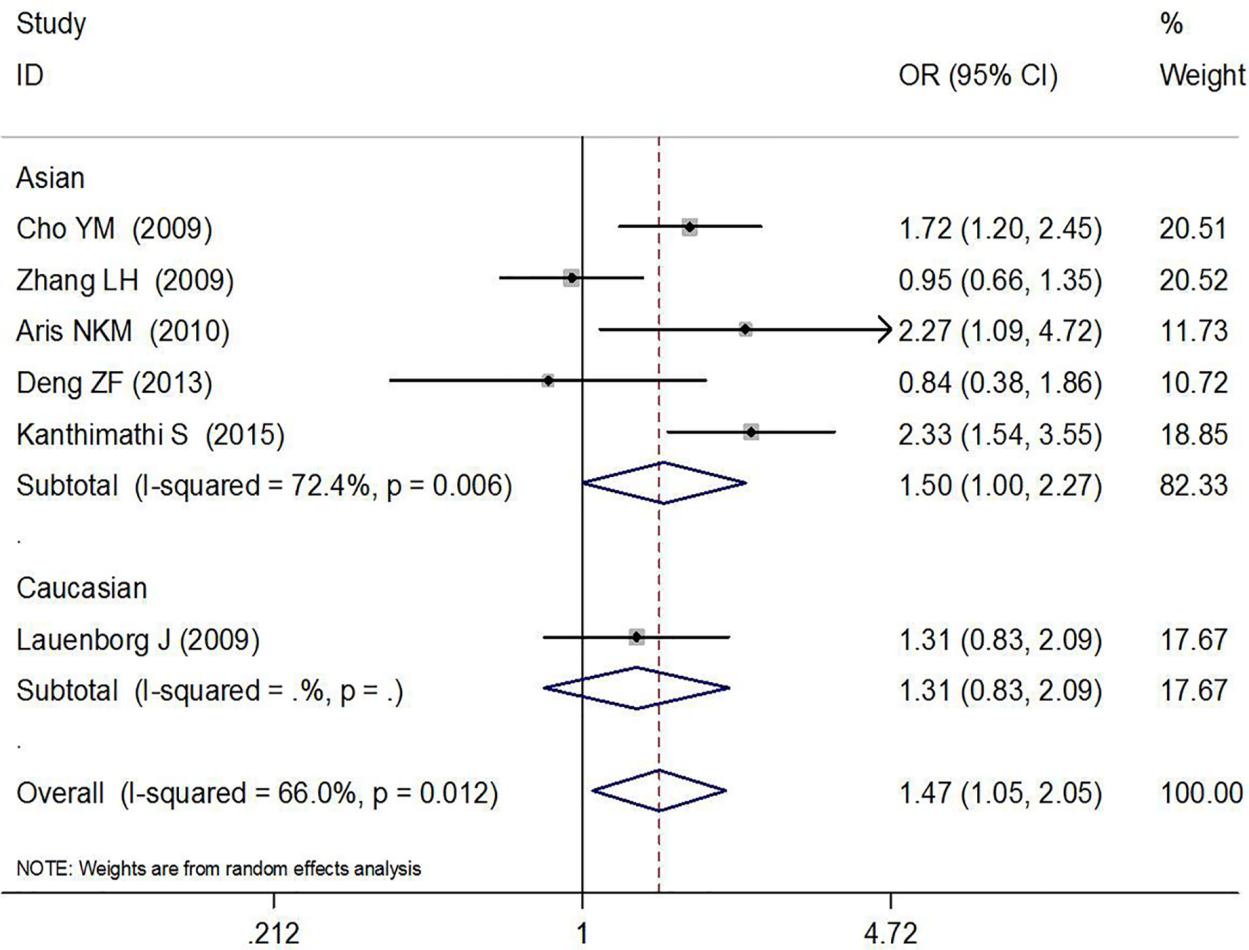
Forest plot risk of GDM associated with the *CDKAL1* rs7756992 (GG *vs.* AA).

In the stratified analysis by ethnicity and sample size (≥200 or <200) of the cases, positive results were found by comparing the variant AG heterozygous and GG homozygous genotypes with the wild-type homozygous AA genotype (AG *vs.* AA: OR = 1.20, 95% CI = 1.03, 1.39, *p* = 0.022 in Asians, OR = 1.35, 95% CI = 1.04, 1.76, *p* = 0.023 in Caucasians; and AG *vs.* AA: OR = 1.22, 95% CI = 1.06, 1.41, *p* = 0.005 and GG *vs.* AA: OR = 1.49, 95% CI = 1.01, 2.19, *p* = 0.044 in the sample size ≥200 group). These results are shown in **Figures 2**, **3** and **Table 2**.

**Table 2:** Meta-analysis of the *CDKAL1* rs7756992 and rs7754840 polymorphisms on the risk of GDM.

Polymorphism	No. of case/control	Heterozygous genotype				Homozygous genotype			
		OR (95% CI)	*p*	*p*_Het_	*I* ^2^	OR (95% CI)	*p*	*p*_Het_	*I* ^2^
**rs7756992 A>G**	2,376/4,458	**1.23 (1.08–1.41)**	**0.002**	0.416	0.0%	**1.47 (1.05–2.05)**	**0.024**	0.012	66.0%
Ethnicity									
Asians	2,101/2,119	**1.20 (1.03–1.39)**	**0.022**	0.361	8.0%	1.50 (1.00–2.27)	0.051	0.006	72.0%
Caucasians	275/2,339	**1.35 (1.04–1.76)**	**0.023**	–	–	1.31 (0.83–2.09)	0.249	–	–
Sample size									
≥200	2,042/4,185	**1.22 (1.06–1.41)**	**0.005**	0.558	0.0%	**1.49 (1.01–2.19)**	**0.044**	**0.009**	73.8%
<200	334/273	1.35 (0.89–2.05)	0.161	0.098	63.4%	1.40 (0.53–3.70)	0.498	0.071	69.3%
**rs7754840 C>G**	4,819/5,873	**1.36 (1.13–1.65)**	**0.002**	<0.001	74.1%	**1.76 (1.37–2.26)**	**<0.001**	<0.001	73.5%
Ethnicity									
Asians	4,254/5,161	**1.45 (1.15–1.83)**	**0.002**	<0.001	79.0%	**1.82 (1.35–2.44)**	**<0.001**	<0.001	79.1%
Caucasians	565/712	1.11 (0.87–1.41)	0.392	0.732	0.0%	1.46 (0.97–2.19)	0.066	0.444	0.0%
Sample size									
≥200	4,490/5,600	**1.21 (1.07–1.37)**	**0.003**	0.119	39.0%	**1.74 (1.33–2.28)**	**<0.001**	<0.001	76.7%
<200	329/273	**3.32 (1.40–7.90)**	**0.007**	0.039	76.6%	2.00 (0.92–4.34)	0.080	–	–

GDM, gestational diabetes mellitus.

Bold values indicate statistically significant values.

### Association Between rs7754840 and Gestational Diabetes Mellitus Risk

The association between rs7754840 and the risk of GDM has been explored in 10 high-quality studies, including 4,819 GDM patients and 5,873 controls (12, 29–37). The results of the meta-analysis showed that compared with the wild-type GG genotype, the variant CG heterozygous and CC homozygous genotypes were significantly associated with increased risks of GDM (CG *vs.* GG: OR = 1.36, 95% CI = 1.13, 1.65, *p* = 0.002; CC *vs.* GG: OR = 1.76, 95% CI = 1.37, 2.26, *p* < 0.001).

**Figure 4:**
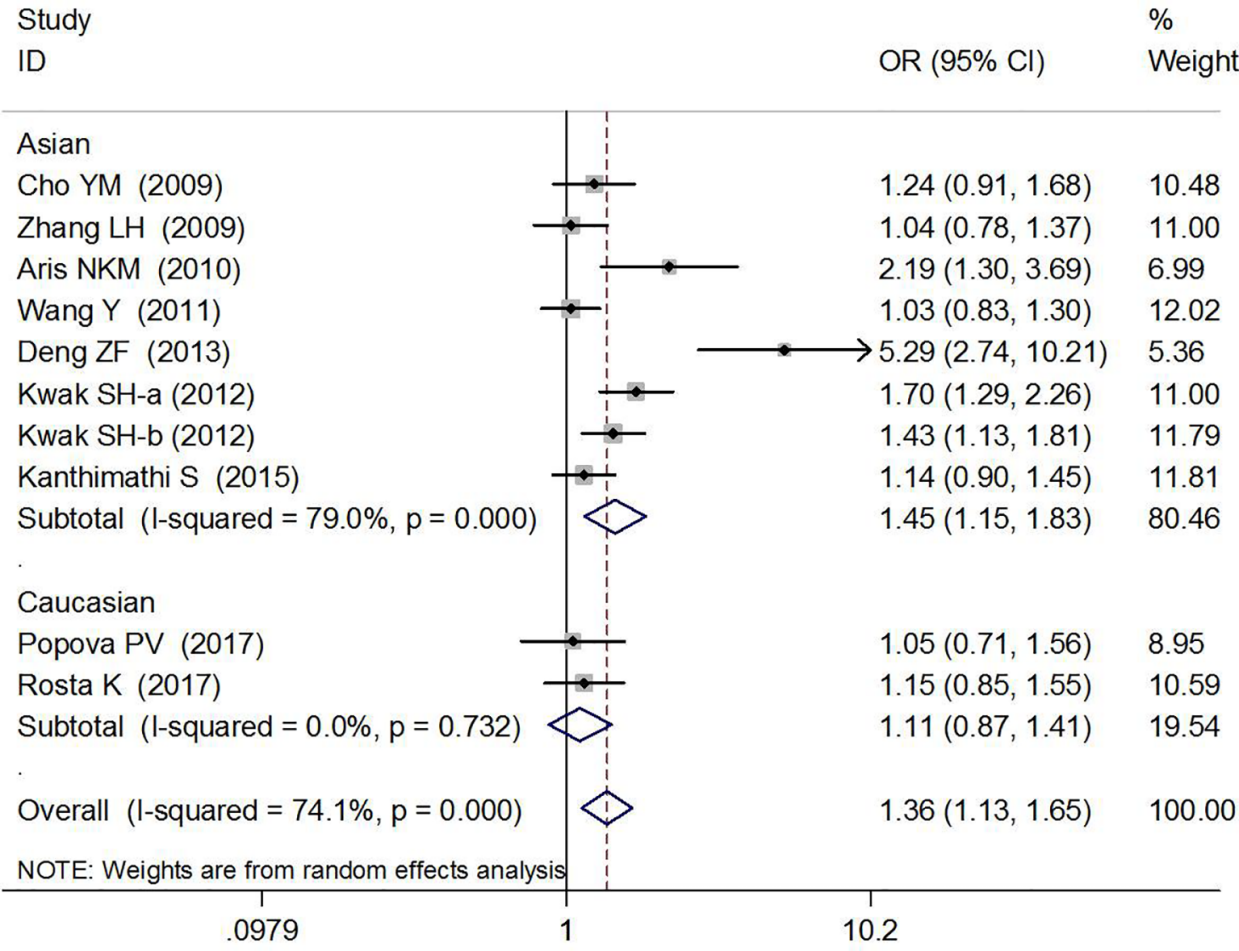
Forest plot risk of GDM associated with the *CDKAL1* rs7754840 (CG *vs.* GG).

**Figure 5:**
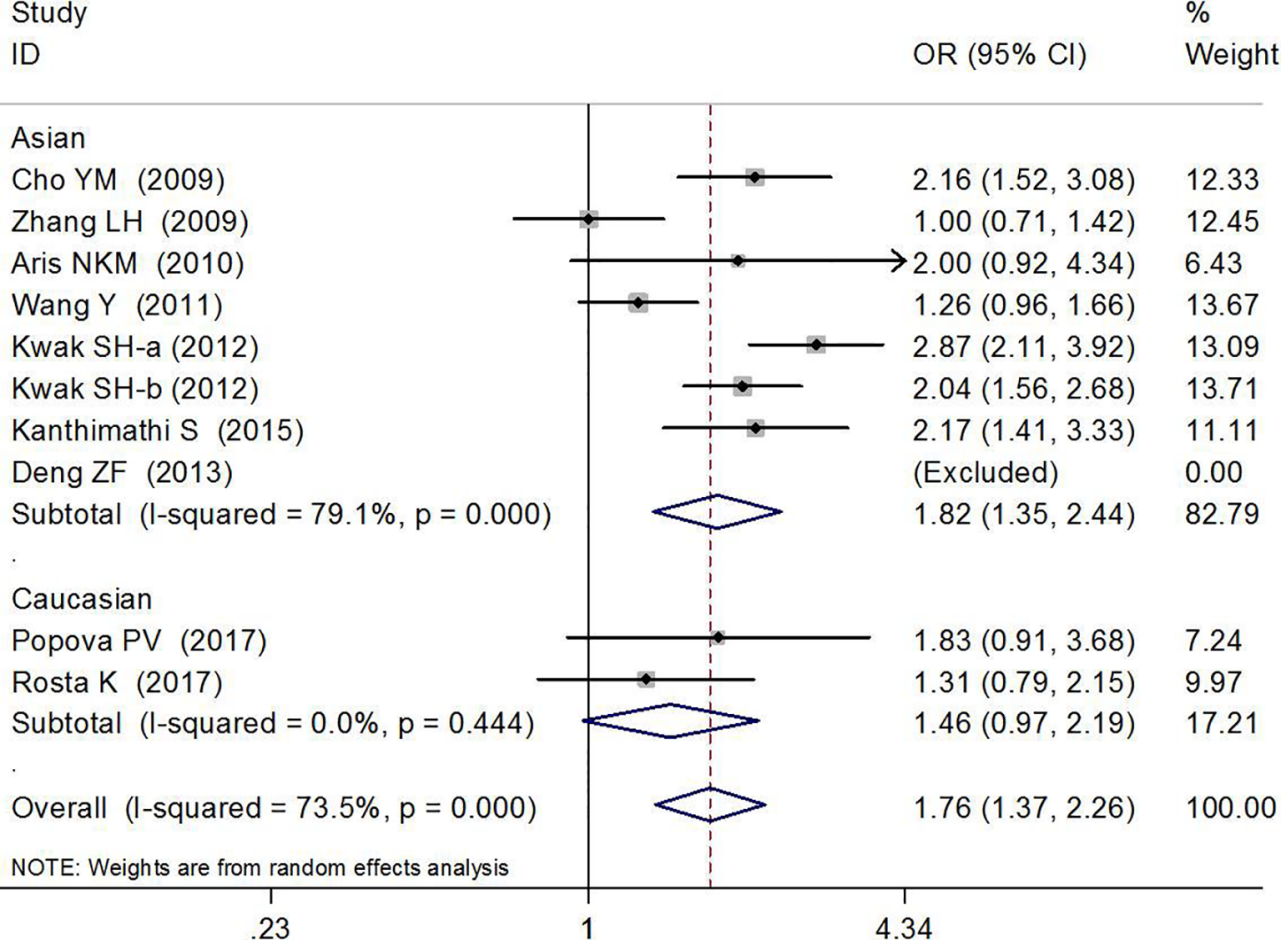
Forest plot risk of GDM associated with the *CDKAL1* rs7754840 (CC *vs.* GG).

The stratified analysis suggested a significant association between rs7754840 and GDM risk (CG *vs.* GG: OR = 1.45, 95% CI = 1.15, 1.83, *p* = 0.002 in Asians, OR = 1.21, 95% CI = 1.07,1.37, *p* = 0.003 in sample sizes ≥200 and OR = 3.32, 95% CI = 1.40, 7.90, *p* = 0.007 in smaller sample size groups; CC *vs.* GG: OR = 1.82, 95% CI = 1.35, 2.44, *p* < 0.001 in Asians and OR = 1.74, 95% CI = 1.33, 2.28, *p* < 0.001 in sample sizes ≥200 group). These results are shown in **Figures 4**, **5** and **Table 2**.

### Evaluation of Heterogeneity

Meta-regression analysis was performed to explore the source of heterogeneity by ethnicity and the sample size of the case group (≥200). In the homozygous genotype comparison of rs7756992, meta-regression analysis showed that heterogeneity was not significantly associated with ethnicity (*t* = 0.27, *p* = 0.797) or sample size (*t* = 0.12, *p* = 0.910). The subsequent leave-one-out analysis showed that after removing the report by Zhang et al. (31), the overall heterogeneity (*p*_heterogeneity_ and *I* ^2^) changed from 0.012% and 66% to 0.135% and 43%.

For the observed significant heterogeneity of rs7754840 and the susceptibility to GDM, meta-regression analyses showed that the sample size (*t* = −2.45, *p* = 0.034) might be the main heterogeneity source in the heterozygote genotype comparison. However, in the homozygous genotype comparison, we did not find ethnicity (*t* = 0.46, *p* = 0.657) or sample size (*t* = 0.21, *p* = 0.836) to be a significant cause of the heterogeneity between studies. In addition, leave-one-out analysis showed that no single factor had a significant association with the observed heterogeneity.

### Publication Bias and Sensitivity Analysis

**Figure 6:**
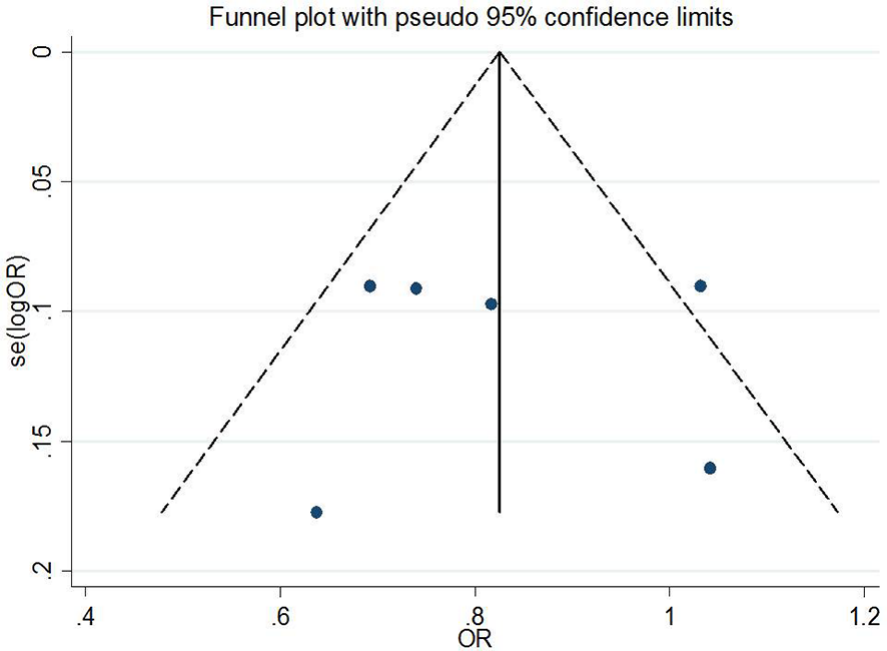
Funnel plot analysis to detect publication bias (rs7756992).

**Figure 7:**
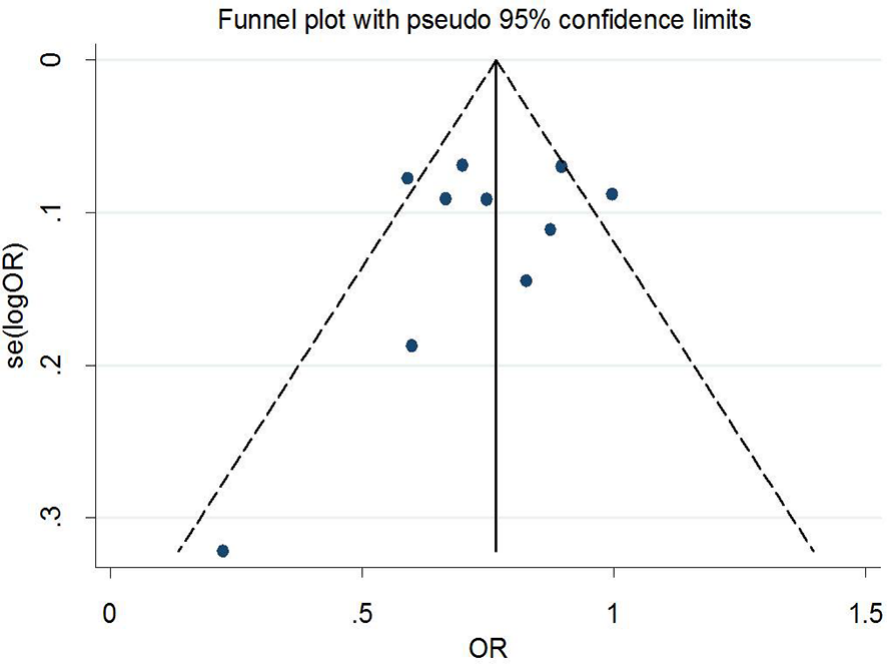
Funnel plot analysis to detect publication bias (rs7754840).

**Figure 8:**
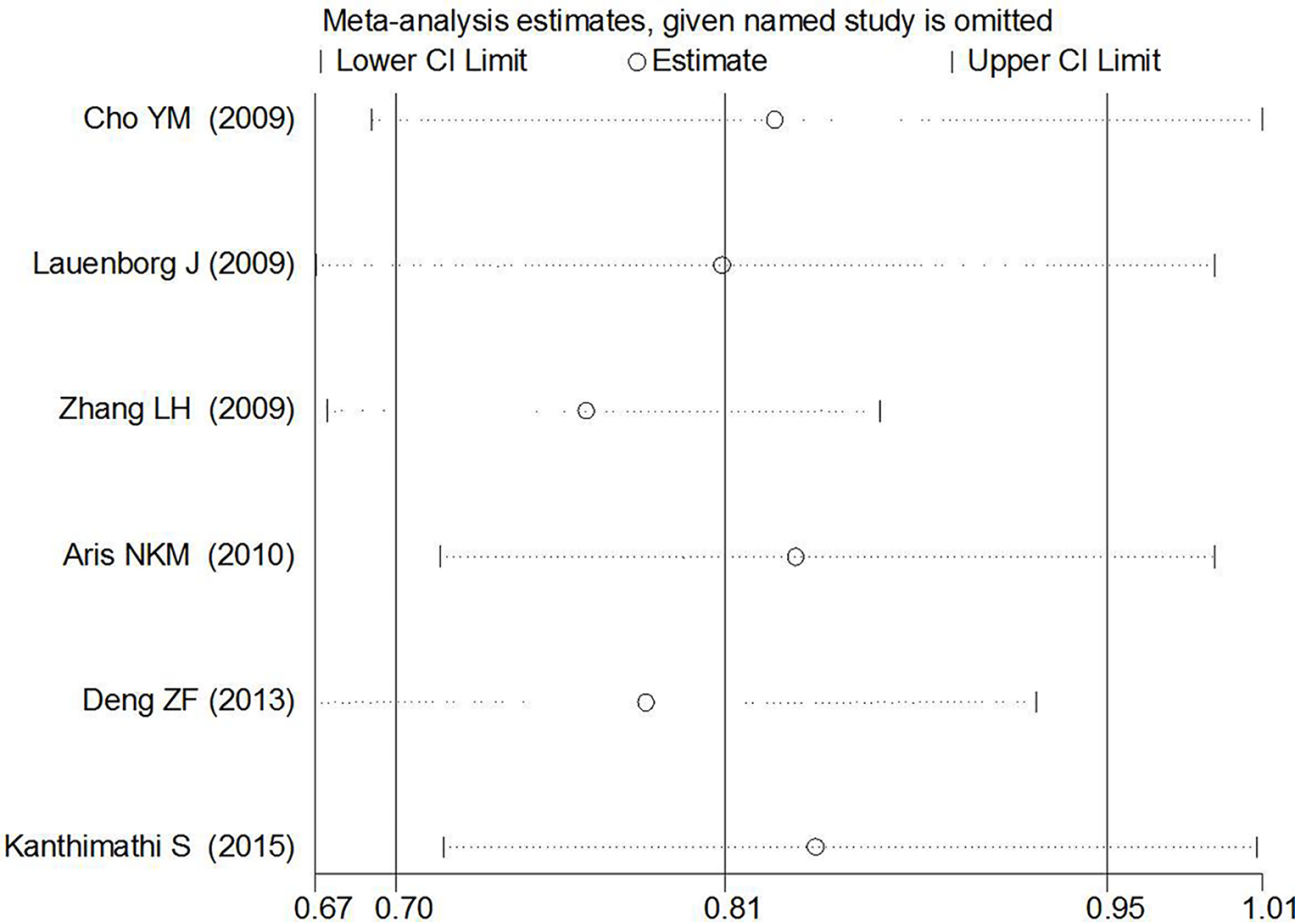
Sensitivity analyses of rs7756992 polymorphism and GDM risk.

**Figure 9:**
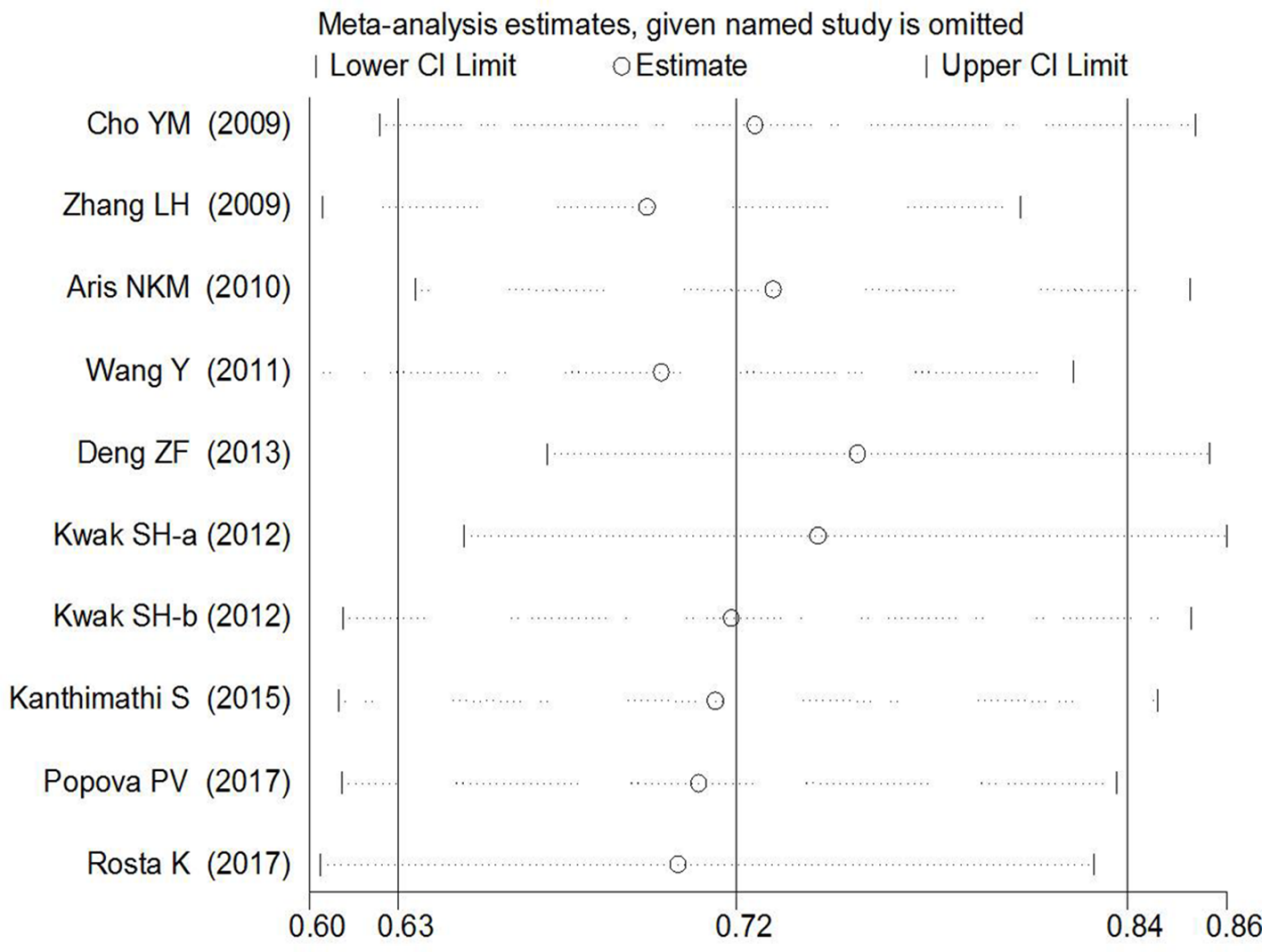
Sensitivity analyses of rs7754840 polymorphisms and GDM risk.

Begg’s tests were performed to identify the publication bias of the included studies, and the shape of the funnel plots did not reveal any evidence of obvious asymmetry (**Figures 6** and **7**). Further statistical analysis suggested no significant statistical findings for the publication bias of rs7756992 (AG *vs.* AA: z = −0.19, *p*_Begg’s_ = 0.851; GG *vs.* AA: z = 0.19, *p*_Begg’s_ = 0.851) and rs7754840 (CG *vs.* GG: z = 1.61, *p*_Begg’s_ = 0.107; CC *vs.* GG: z = 0.31, *p*_Begg’s_ = 0.754) in the present study. Sensitivity analyses suggesting no single study significantly changed the pooled ORs (**Figures 8** and **9**).

### False-Positive Report Probability Analysis

The FPRP was adopted to assess the noteworthiness of the significant associations between the studied rs7756992 and rs7754840 polymorphisms and the risk of GDM. At the prior probability of 0.1 and a relatively stringent FPRP cutoff value of 0.2, the FPRP values calculated for the positive findings were 0.018 (overall), 0.043 (sample size ≥200), and 0.165 (Asian group) for rs7756992 A>G, and 0.021 (overall), 0.026 (sample size ≥200), and 0.028 (Asian group) for rs7754840 C>G in heterozygous genotype comparisons; in addition, FPRP values were 0.013 (overall), 0.017 (sample size ≥200), and 0.022 (Asian group) for rs7754840 C>G in homozygous genotype comparisons. These FPRP values suggested that the above positive findings were probability correct and reliable (**Table 3**).

**Table 3:** FPRP analysis for the significant associations of the *CDKAL1* genetic variations and GDM risk.

Comparison group	Study model	OR (95% CI)	Prior probability					
			0.25	0.1	0.01	0.001	0.0001	0.00001
**rs7756992 A>G**								
Overall	GA *vs.* AA	1.23 (1.08–1.41)	0.006	**0.018**	0.166	0.667	0.953	0.995
	GG *vs.* AA	1.47 (1.05–2.05)	0.115	0.281	0.811	0.977	0.998	1.000
Sample size ≥ 200	GA *vs.* AA	1.22 (1.06–1.41)	0.015	**0.043**	0.332	0.834	0.980	0.998
	GG *vs.* AA	1.49 (1.01–2.19)	0.203	0.432	0.893	0.988	0.999	1.000
Asian	GA *vs.* AA	1.20 (1.03–1.39)	0.062	**0.165**	0.686	0.957	0.995	1.000
Caucasian	GA *vs.* AA	1.35 (1.04–1.76)	0.083	0.213	0.748	0.968	0.997	1.000
**rs7754840 C>G**								
Overall	CG *vs.* GG	1.36 (1.13–1.65)	0.007	**0.021**	0.190	0.702	0.959	0.996
	CC *vs.* GG	1.76 (1.37–2.26)	0.004	**0.013**	0.129	0.600	0.937	0.993
Sample size ≥ 200	CG *vs.* GG	1.21 (1.07–1.37)	0.009	**0.026**	0.229	0.750	0.968	0.997
	CC *vs.* GG	1.74 (1.33–2.28)	0.006	**0.017**	0.160	0.658	0.951	0.995
Sample size < 200	CG *vs.* GG	3.32 (1.40–7.90)	0.359	0.627	0.949	0.995	0.999	1.000
Asian	CG *vs.* GG	1.45 (1.15–1.83)	0.009	**0.028**	0.240	0.761	0.970	0.997
	CC *vs.* GG	1.82 (1.35–2.44)	0.008	**0.022**	0.201	0.718	0.962	0.996

FPRP, false-positive report probability; GDM, gestational diabetes mellitus.

Bold values indicate statistically significant values.

### Trial Sequential Analysis

**Figure 10:**
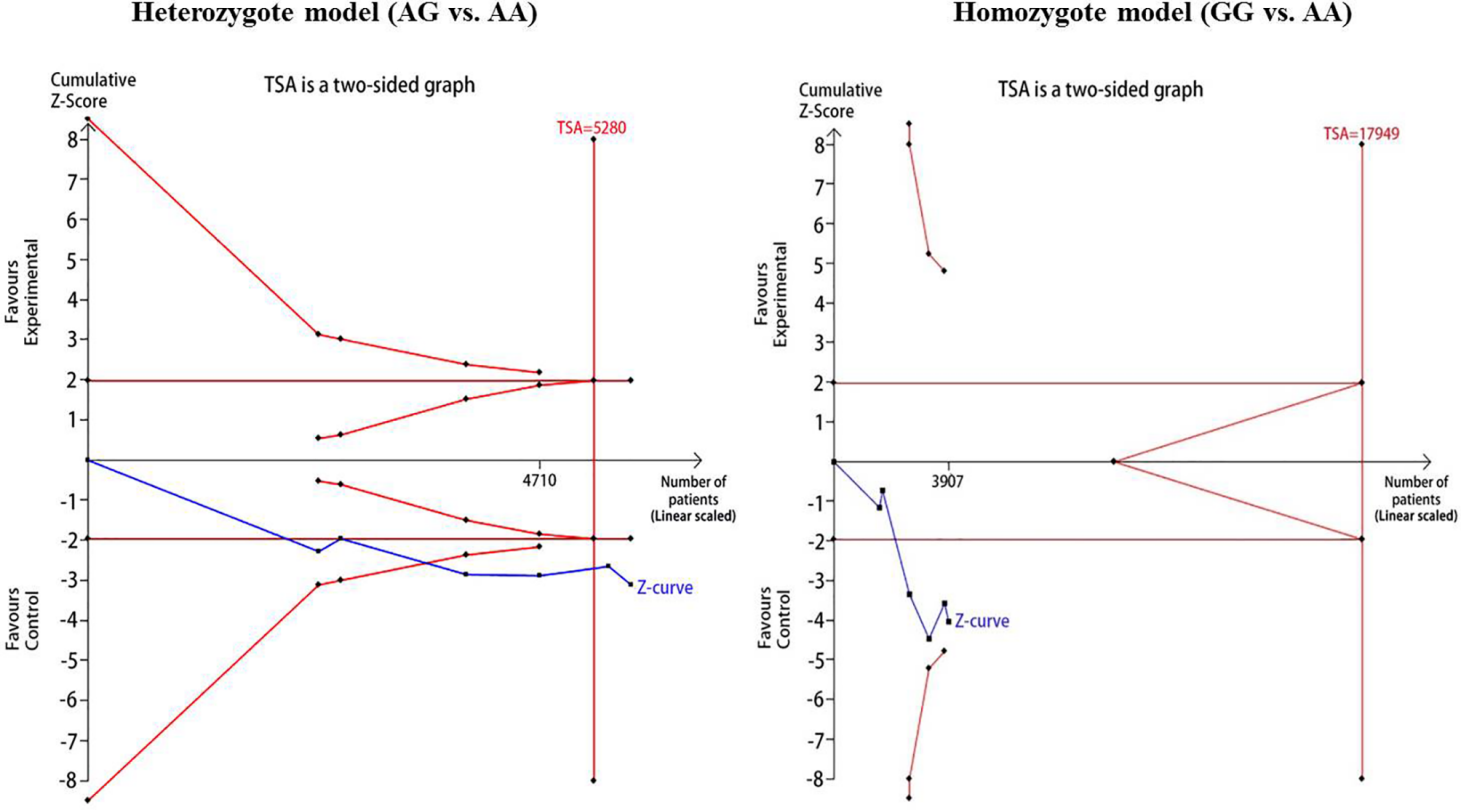
TSA for *CDKAL1* rs7756992 A>G. We calculated α-spending adjusted required information size (RIS) by using α = 0.05 (two-sided), power = 80%. The cumulative Z-curve (Blue); Conventional boundary (Deep red); TSA boundary (red).

**Figure 11:**
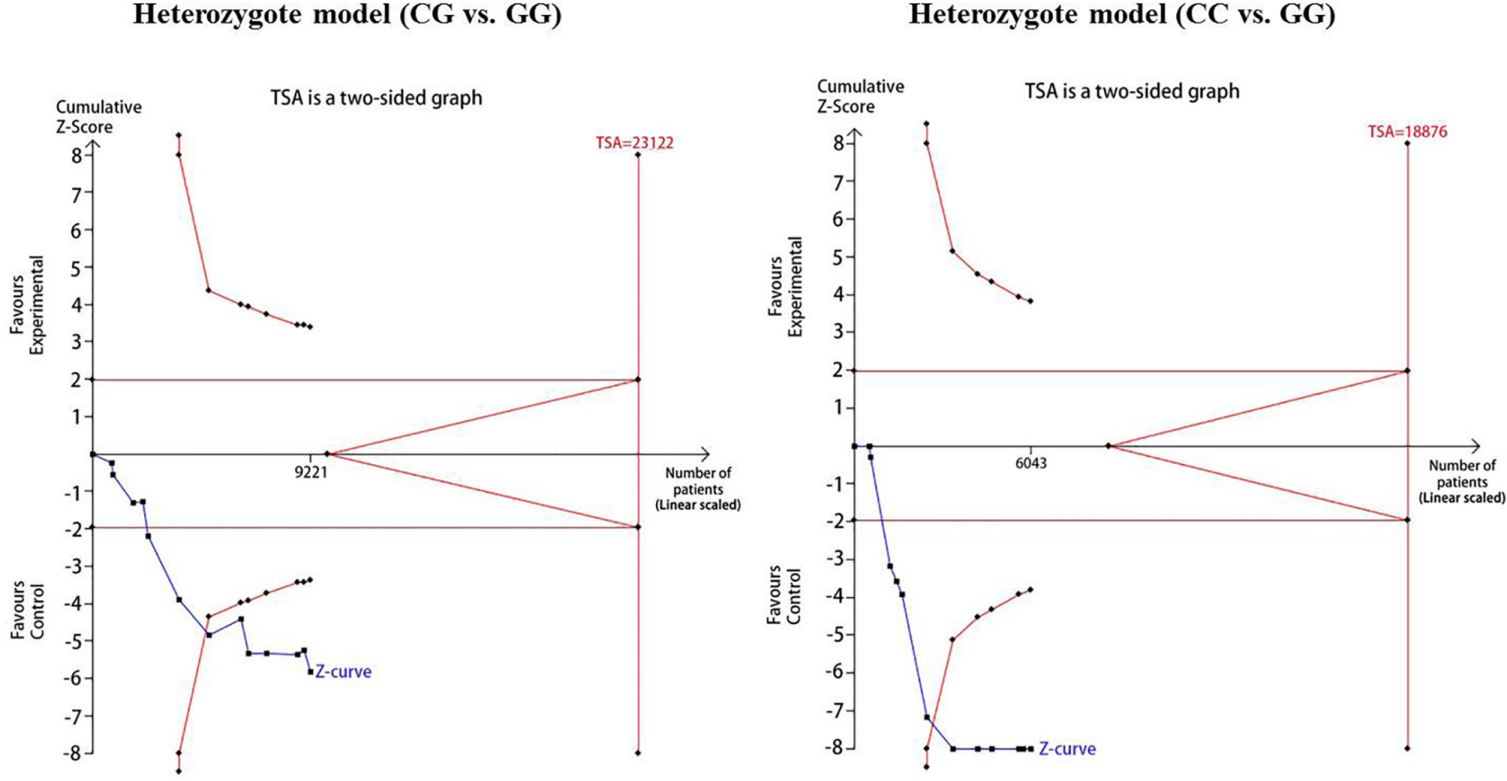
TSA for *CDKAL1* rs7754840 C>G.

For reducing the random errors and increasing the credibility of the conclusions, TSA was performed. It showed that the cumulative Z-curve crossed both the conventional cutoff value and the TSA boundaries, suggesting that the accumulated amount information was sufficient and that no more study evidence was needed for the significant association of *CDKAL1* rs7756992 and the risk of GDM under the heterozygote model (AG *vs.* AA). However, in the homozygote model (GG *vs.* AA), the cumulative Z-curve crossed only the conventional boundary but did not cross the TSA boundary, indicating that additional studies were necessary (**Figure 10**). Meanwhile, the TSA confirmed a significant association between rs7754840 and the susceptibility to GDM under the heterozygote model (CG *vs.* GG) and homozygote models (CC *vs.* GG) because the cumulative Z-curve crossed both the conventional cutoff value and TSA boundaries (**Figure 11**). Even if the cumulative amount information did not reach the RIS, no additional study evidence was needed to verify the conclusions.

## Discussion

GDM is a special form of diabetes that occurs during pregnancy (32). Current studies have demonstrated that gene polymorphisms might help to provide insight on the underlying backgrounds of complex diseases. Hence, many candidate gene polymorphisms, including *CDKAL1* rs7756992 and rs7754840, have been tested for a relationship with genetic susceptibility to GDM. Some studies demonstrated a significant susceptibility to GDM associated with rs7756992 and rs7754840 in allele models (25, 29, 32). However, Zhang et al. (31) and Deng et al. (34) found negative associations between rs7756992 and the risk of GDM. Several studies concluded that there was no association between rs7754840 and the risk of GDM (28, 36, 37). The present meta-analysis summarizes the evidence to date on the association between *CDKAL1* rs7756992 and rs7754840 and the risk of GDM.

The meta-analysis results indicated that the variant heterozygous and homozygous genotypes of the two polymorphisms were associated with increased GDM risk. Results of TSA suggested that the accumulated amount of information was sufficient and that no additional studies were required to demonstrate the significant association of *CDKAL1* rs7756992 and the risk of GDM under the heterozygote model but not in the homozygote model. The TSA also confirmed the significant association between rs7754840 and the susceptibility to GDM under the heterozygote and homozygote models without further study evidence. The study by Zhang et al. (31) might be the main source of heterogeneity in the homozygous genotype comparison of rs7756992, and “sample size” might significantly influence the heterogeneity in the heterozygote genotype comparison of rs7754840. No significant statistical publication bias was detected for the analyses of candidate SNPs and GDM risk. The findings support that the *CDKAL1* polymorphisms modify the susceptibility of pregnant women to GDM.

GDM is characterized by insulin resistance and impaired insulin secretion (38). The variants of *CDKAL1* may influence *CDKAL1* expression and leading to impaired β-cell function and insulin secretion. A study of Steinthorsdottir et al. (39) suggested that the *CDKAL1* intron variant rs7756992 was associated with insulin response. A study by Kwak et al. (12) showed that *CDKAL1* rs7754840 played a role in decreasing fasting insulin concentration and homeostasis model assessment for β-cell function (HOMA-β). One study has shown that variants located at the gene intron region mainly inactivate the splice-donor site and change the splicing pattern of pre-mRNA (40). The splicing error may lead to a wrong transcript product and ultimately influence gene function. However, to date, there are still no relevant studies interpreting how the studied two loci affect CDKAL1 function and change susceptibility to GDM. Therefore, further studies of the biological function of the two polymorphisms in the etiology of GDM are warranted.

Previous meta-analysis showed that polymorphisms located on *CDKAL1* gene were significantly associated with genetic susceptibility to GDM (41, 42). Guo et al. (41) found that the variants rs7756992 and rs7754840 showed significant correlation with GDM risk under the allele, recessive, dominant, homozygote, and heterozygote models. The study of Mao et al. (42) indicated that *CDKAL1* rs7754840 was associated with GDM risk among East Asians. However, if the number of participants was less than required, based on a realistic intervention effect, the constant application of a traditional 95% CI or 5% statistical significance threshold will lead to a false-positive or false-negative conclusion. Thus, in the present trial sequential meta-analysis, we confirmed the association between *CDKAL1* polymorphisms rs7756992 or rs7754840 with the risk of GDM under the allele models, and no more samples are needed to further evaluate these findings.

The FPRP analysis is an effective approach to verify the noteworthiness of significant association findings. For the present study, a relatively stringent FPRP threshold of 0.2 was set, and much lower FPRP values of the observed significant associations between rs7756992 G>A and rs7754840 C>G variations, and the risk of GDM suggested that the positive findings were probability accurate and reliable. Hence, we believe that the association of the two variations and GDM risk is credible.

Some limitations in the present meta-analysis should be pointed out. First, the literatures included in this meta-analysis are retrospective studies. Compared with prospective studies, the research data are vulnerable to selection bias and recall bias. Second, only published data were included, and some of the sample sizes were relatively small. These may affect the overall effect assessment between studied polymorphisms and GDM. To ensure the reliability of the results, a TSA was performed, and FPRP values of note were assessed. Third, this meta-analysis simply evaluated the association between rs7756992 or rs7754840 genotypes and GDM risk without considering the effects of other genetic markers or environmental factors (43, 44). If individual-level data were available, a much more precise analysis to identify the interactions between gene–gene and gene–environment could also have been done.

In summary, the present study supports the significant association of the *CDKAL1* polymorphisms rs7756992 and rs7754840 with an increased risk of GDM. Further functional studies are warranted to clarify the potential mechanism in the pathogenesis of GDM.

## Data Availability Statement

The original contributions presented in the study are included in the article/supplementary material. Further inquiries can be directed to the corresponding authors.

## Author Contributions

X-YY and D-BL: protocol/project development and manuscript editing. D-BL and L-PS: data collection and analysis and manuscript writing. S-DW: data analysis and manuscript writing. X-LW: data collection and management. All authors contributed to the article and approved the submitted version.

## Funding

This study was supported by Basic ability improvement project of young and Middle-aged College Students in Guangxi (2020KY2028), the National Natural Science Foundation of China (81760614), the Key R&D Program (2018AB62004), and College Students’ Innovation Project (201710601103, 201810601031).

## Conflict of Interest

The authors declare that the research was conducted in the absence of any commercial or financial relationships that could be construed as a potential conflict of interest.

## Publisher’s Note

All claims expressed in this article are solely those of the authors and do not necessarily represent those of their affiliated organizations, or those of the publisher, the editors and the reviewers. Any product that may be evaluated in this article, or claim that may be made by its manufacturer, is not guaranteed or endorsed by the publisher.
